# Perceived Object Stability Depends on Multisensory Estimates of Gravity

**DOI:** 10.1371/journal.pone.0019289

**Published:** 2011-04-27

**Authors:** Michael Barnett-Cowan, Roland W. Fleming, Manish Singh, Heinrich H. Bülthoff

**Affiliations:** 1 Department of Human Perception, Cognition and Action, Max Planck Institute for Biological Cybernetics, Tübingen, Germany; 2 Department of Psychology, Rutgers University Center for Cognitive Science, Piscataway, New Jersey, United States of America; 3 Department of Brain and Cognitive Engineering, Korea University, Seoul, Korea; University of Regensburg, Germany

## Abstract

**Background:**

How does the brain estimate object stability? Objects fall over when the gravity-projected centre-of-mass lies outside the point or area of support. To estimate an object's stability visually, the brain must integrate information across the shape and compare its orientation to gravity. When observers lie on their sides, gravity is perceived as tilted toward body orientation, consistent with a representation of gravity derived from multisensory information. We exploited this to test whether vestibular and kinesthetic information affect this visual task or whether the brain estimates object stability solely from visual information.

**Methodology/Principal Findings:**

In three body orientations, participants viewed images of objects close to a table edge. We measured the critical angle at which each object appeared equally likely to fall over or right itself. Perceived gravity was measured using the subjective visual vertical. The results show that the perceived critical angle was significantly biased in the same direction as the subjective visual vertical (i.e., towards the multisensory estimate of gravity).

**Conclusions/Significance:**

Our results rule out a general explanation that the brain depends solely on visual heuristics and assumptions about object stability. Instead, they suggest that multisensory estimates of gravity govern the perceived stability of objects, resulting in objects appearing more stable than they are when the head is tilted in the same direction in which they fall.

## Introduction

**Figure 1:**
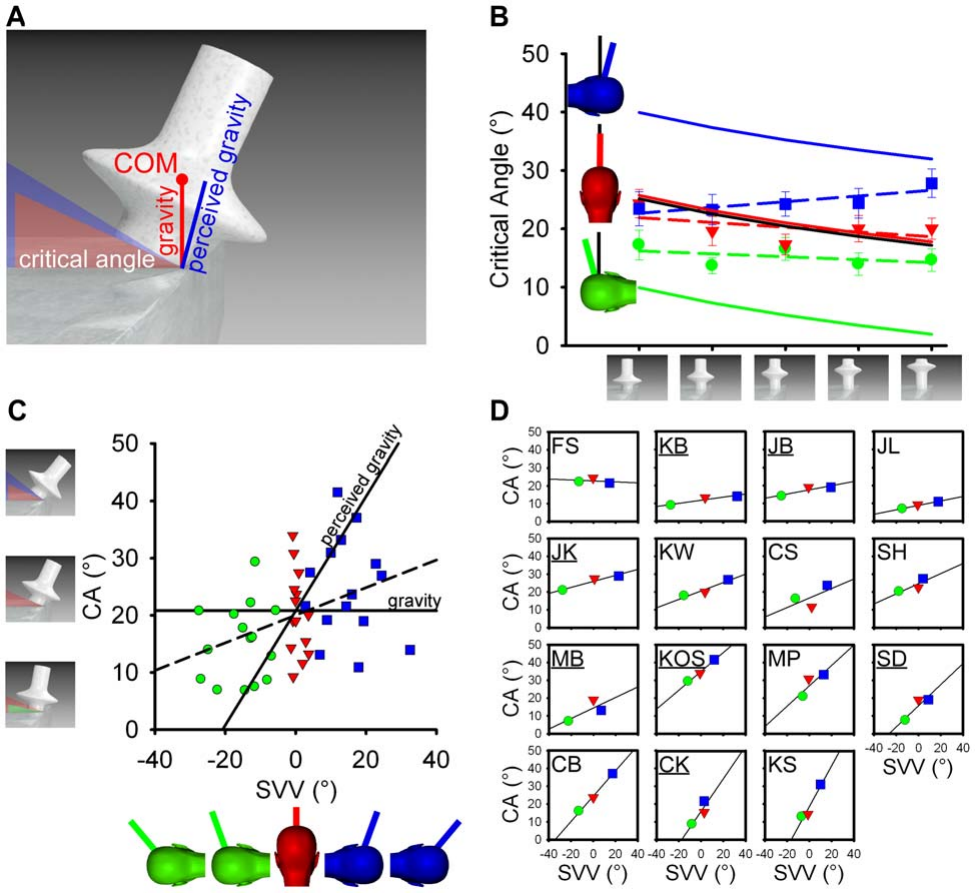
Influence of body tilt on perceived object stability. (**A**) Stimuli. Critical angle (CA) predictions (shaded areas) relative to physical (red) and perceived (blue) gravity. (**B**) Results. Mean CA when upright (▾), left (•) and right side down (▪) for objects with a different COM relative to physical (black) and perceived (coloured solid lines) gravity. Linear regression slopes are shown as coloured dashed lines. Error bars are ±1 S.E. Cartoon inserts indicate the extent to which the SVV shifts towards the body. (**C**) Correlation (dashed line) between the SVV and the perceived CA averaged across all objects. Here the perceived gravity prediction is based on the average SVV setting and the physical gravity prediction is based on the ground truth CA averaged across the five objects. (**D**) Correlations between the CA and the SVV for each participant ordered according to the CA-SVV slope. Underlined initials identify control experiment participants.

An object's perceived stability affects our interactions with it and our expectations about its behaviour [1], [2]. In order to know whether an object will fall over or right itself, the brain must accurately represent the physical laws governing object stability. When an object is in a uniform gravitational field, all forces acting on the object can be represented by a single resultant force and the point at which this resultant force acts is called the centre of mass. In accordance with Newton's first and second laws of motion [3] when the gravity-projected centre-of-mass (COM) of an object lies directly above the point or area of support, there is no net torque and the object remains in static equilibrium. We will call the critical angle (CA) the angle through which the object must be rotated so that it corresponds to the situation when the centre of mass is vertically above the point of support (see red gravity projection and red shaded area in Figure 1A). When the COM lies outside the support area (> CA), the object falls over. When the COM lies inside the support area (< CA), the object rights itself. Thus an object at the edge of a table whose COM is high (i.e., top-heavy) will sooner fall off the table than an object whose COM is lower in the presence of a perturbation.

The force of gravity is not sensed directly. It is the indirect effects of gravity that are detected. To estimate an object's stability visually, the brain must integrate information across the shape to estimate the COM position relative to the support point and compare its orientation to gravity [4]. While it has been shown that observers typically underestimate the CA, suggestive of a conservative bias to not allow objects to fall [4], whether the brain relies solely on visual heuristics to estimate object stability has not previously been investigated. When observers lie on their sides, gravity is perceived as tilted towards the orientation of the body [5]–[12], consistent with a representation of gravity derived from multisensory information [11]–[15]. We exploited this to determine whether CA estimates are consistent with gravity's true direction or the direction in which gravity is perceived.

The perceived direction of gravity can be measured using the subjective visual vertical (SVV) [5]–[10]. If objects are perceived to topple over when the *perceived gravity*-projected COM lies outside the support area then when a participant lays right side down (RSD) the CA of a rightward leaning object should increase compared to when they are upright (Figure 1A, blue line and shaded area). Likewise the CA should decrease when lying left side down (LSD). The extent to which the CA changes with body posture, compared to how the SVV changes, provides a metric for assessing the contribution of multisensory estimates versus purely visual estimates of gravity in determining the perceived stability of objects.

By dissociating biased from veridical estimates of gravity we show that the perceived critical angle is significantly biased in the same direction as the subjective visual vertical, indicating that a multisensory estimate of gravity is used when judging whether objects will fall or not.

## Results

Participants either sat upright or lay on their left or right side and viewed stimuli presented on a laptop computer through a circular tube and responded with button presses. In one task, participants viewed computer rendered images of objects with different mass distributions depicted close to the precipitous right edge of a table (see Figure 1B and Materials and Methods). The vertical direction in the depicted scene was aligned with the direction of gravity in the participant's physical environment and the table was always upright in the real world and therefore provided a visual reference to earth-horizontal. In a second task the perceived direction of gravity was measured using the SVV where participants indicated whether a visual line was oriented clockwise or counterclockwise relative to gravitational vertical.

The SVV results show that gravity is perceived veridically when upright (0.6°, SE: 0.4) but is perceived as tilted towards the body (Figure 1B) when left (−15.2°, SE: 1.8) and right side down (14.8°, SE: 2.1; F(1.2,16.7) = 70.3, p<.001). The perceived CA is influenced by body orientation (F(2,28) = 22.5, p<.001) such that left tilted participants underestimate – and right tilted participants overestimate – the stability of rightward falling objects in the same direction as the SVV.

This close relation between the perceived CA and the average SVV is highly significant (slope  = .24, r = .40, p = .007; Figure 1C) confirming that participants do not judge the stability of falling objects relative to a veridical estimate of gravity. Rather, the perceived stability of objects is affected by multisensory estimates of gravity's direction. This effect is also found for leftward falling objects and with different background images (see below). Note that while the slope of the relation between the CA and the SVV varies across individuals (Figure 1D), the perceived CA changes in the same direction as perceived gravity in all but one participant - who does estimate object stability relative to an unbiased estimate of gravity's direction.

Body orientation also affects the extent to which object shape influences the perceived stability of objects (F(8,112) = 3.3, p = .002) such that the effect of object shape is most pronounced in the RSD condition. It is not readily apparent what can account for the significant interaction between body orientation and object shape. Given that different frames of reference can influence the perceptual organization of shapes [16]–[21], it seems plausible that participants attend to different aspects of the geometry depending on the object's orientation relative to their body, leading to different response criteria.

Slopes of linear regression fits to the CA of the five objects are significantly shallower than the slope of the ground truth prediction when LSD (t(14) = 3.2, p = .007), upright (t(14) = 2.6, p = .020) and RSD (t(14) = 5.2, p<.001), indicating that top heavy objects are perceived as more stable than they are. In addition, no significant downward shift of CA estimates was found when upright (t(14) = 1.5, p = .15) relative to the ground truth prediction, which would have indicated being conservative in estimating object stability. While CA estimates are significantly down shifted in the LSD condition (t(14) = 4.4, p = .001) and in the RSD condition (t(14) = 1.6, p = .14), these results are difficult to interpret in terms of being conservative given the interaction of the CA with body orientation.

### Leftward Falling Objects

**Figure 2:**
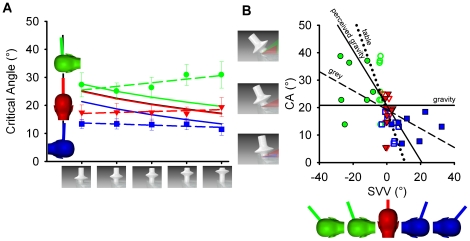
Control experiment results. (**A**) Mean CA for leftward falling objects relative to physical (black) and perceived (coloured lines) gravity as measured with the upright table visual background. (**B**) Correlations between the SVV with the grey background (filled symbols, dashed line), and the SVV with the table image (empty symbols, dotted line) paired with the perceived CA averaged across all objects. Data in **A** and **B** are from the same group of 7 participants from the original group of 15 for two different control experiments. Note that the prediction line for perceived gravity is a negative slope for leftward falling objects and a positive slope for rightward falling objects as shown in Figure 1C. All other conventions as in Figure 1.

An additional perceived CA experiment using 7 of the same participants from the initial experiment was run in order to further study the effect of body posture on the CA. The methods are the same as in the perceived CA experiment above, but here objects are placed close to the precipitous left edge of a table. When comparing estimates for leftward versus rightward falling objects we do not find a significant effect of the direction in which objects fall (F(1,6) = .04, p = .85). Otherwise the results agree completely with those from the original experiment such that body orientation (F(2,12) = 5.3, p = .022) and object shape (F(4,24) = 4.8, p = .005) significantly affect the perceived CA (Figure 2A). Here, however, we find that left tilted participants overestimate – and right tilted participants underestimate – the stability of leftward falling objects in the same direction as the SVV.

### Subjective Visual Vertical (Table Image)

An additional SVV experiment using 7 of the same participants from the initial experiment (the same 7 who participated in the additional perceived CA experiment above) was run in order to determine the effect of visual cues to orientation present during the perceived critical angle experiment (e.g. the table top). The methods are the same as in the SVV experiment above, but here the SVV probe is superimposed on the image of the upright table used previously. Here, if a participant lying right side down integrates visual information about gravity's direction then the SVV should be less affected by body orientation, than found previously for the grey background. Further, if the stability of a leftward falling object is judged in accordance with a biased rather than a veridical perception of gravity, then the CA should decrease by the amount of SVV shift compared to the ground truth. Likewise the CA should increase by the amount of SVV shift when lying left side down.

The SVV results show that gravity is perceived as veridical when upright (−0.2°, SE: 0.5) and there is a significant effect of body orientation (F(1.2,16.7) = 70.3, p<.001) where gravity is perceived as tilted towards the body when left (−3.8°, SE: .15) and right side down (2.5°, SE: 1.4). A significant interaction between visual background (grey, image) and body posture (LSD, upright, RSD; F(1.1,6.6) = 19.0, p<.01) confirms that the presence of the visual background significantly reduces the extent to which gravity is perceived as shifted towards the body by a factor of 78.4%. Despite this reduced effect of perceived gravity shifting towards the body, a significant negative correlation (slope = −1.7, r = −.64, p = .002) between the perceived CA and the SVV (Figure 2B) confirms that participants use perceived gravity as a frame of reference when judging the stability of leftward falling objects.

## Discussion

Humans spend most of their time engaging in the world with an upright posture. Here sensory information about self-orientation is usually redundant and the perceived stability of objects [2] and the body [5]–[10] are generally veridical. Knowing an object's physical stability is important as it affects our interactions with it and our expectations about its behaviour [1], [2]. Equally important is knowing about the orientation and stability of the body, which affects our ability to coordinate our actions [12]–[15], [22], [23], maintain our balance [24] and correctly identify objects [8]–[10]. The vestibular system, which detects tilt of the head relative to gravity, provides a more reliable signal for small tilts of the head relative to an upright posture than for large tilts of the head [5]. Our results are in accord with previous studies showing that the perceived direction of gravity is influenced by visual, body sense and prior information because of compensation for poor vestibular sensitivity when tilted which helps maintain optimal perception and action [5]–[12], [25]–[27].

It has been suggested that multisensory information is integrated by the brain to generate separate but related frames of reference tailored for different task demands [8]–[12]. The present study extends and qualifies these previous results by showing that the stability of objects is not perceived relative to a veridical estimate of gravity's true direction. Rather, a potentially biased internal representation of gravity derived from multisensory information is used as a frame of reference when estimating the critical angle and the SVV. This is surprising given that the table in the scene provides a strong, purely visual frame of reference that the visual system could use for estimating the gravity direction and computing stability. As both the SVV and CA tasks require comparing a visual object with an unseen line representing gravity's orientation relative to the body, and both tasks relate to stability – of the self and of objects, respectively – we propose that a common internal representation of gravity is used as a frame of reference for both tasks. In agreement with previous studies [25]–[27], we suggest that use of this frame of reference is optimized for when the body is upright at the cost of introducing systematic errors in visual estimates of physical stability when tilted; objects appear more stable than they are when the head is tilted in the same direction in which they fall.

Humans tend to adopt a reasonable strategy of using the perceived centre of an object's shape, which is close to the centre of mass, to determine an object's centre of mass [4], [28]–[30]. This strategy is reasonable in so far as the object is of uniform density. It is important to note that although we explicitly instructed our participants to treat the objects that we used as being of uniform density, biases from this assumption could explain the fact that the effect of object shape on the critical angle did not always follow the physical predictions. Further, it has been shown previously that centre of mass estimates can be inconsistent with stability estimates [4]. Finally, upright observers tend to underestimate the critical angle suggesting a conservative bias to not allow the object to fall [4]. While this hypothesis has strong ecological appeal, it is clear that this conservative tendency was not consistent across all conditions and object shapes used here.

An optimal estimate of gravity's direction is internally represented by the brain to disambiguate [13] or supplement sensory information [15]. Our findings indicate that although the physical laws governing object stability are reasonably accurately represented by the brain, they are in turn biased by multisensory estimates of gravity. This result has important implications for existing theories of how humans perceive the stability of objects. For example, since the work of Piaget [1] it has been shown that children [1], [31] and adults [2] have difficulty in solving problems involving the physical laws which govern equilibrium, even when these laws are explicitly taught to them [32]. We suggest that having to integrate multisensory information, which has been shown to change during development [33], [34], with sex of the participant [9], [34]–[37], and in patients with neuropsychiatric disorders [10], [38]–[40], may contribute to the errors associated with solving these problems.

## Materials and Methods

### Ethics Statement

This research was performed in accordance with the ethical standards specified by the 1964 Declaration of Helsinki and the ethics review board of the Max Planck Institute for Biological Cybernetics which approved this study. All participants gave their informed and written consent prior to their inclusion in the study.

### Participants

14 German diploma students visiting the Max Planck Institute for Biological Cybernetics and one author (MB-C) participated in the study (mean age 25 years; SD = 4.48). All had normal or corrected to normal vision and reported no history of vestibular dysfunction.

### Convention

All orientations are reported with respect to the direction of gravity (0°). Clockwise tilts in roll are assigned positive values, counter-clockwise, negative values.

### Apparatus

Participants either sat upright or lay on foam padding on their left or right side with their head supported by foam blocks to ensure that the head was at 90° relative to gravity. Participants viewed stimuli presented in the fronto-parallel plane on an Apple MacBook Pro 17” laptop computer with a resolution of 53 pixels/cm (32 pixels/°). Peripheral vision was masked to a circular screen of diameter 36° by viewing through a circular tube that also maintained the viewing distance at 30 cm.

### Stimuli and procedure for determining the perceived critical angle

Five objects with a different centre-of-mass (COM) were created as surfaces of revolution using Bernstein polynomials for the longitudinal profile. Varying the parameters of the polynomial shifts the mode of the function without changing the area under the curve, thus preserving object volume while adjusting COM height. A short line segment was added to the bottom end of the curve to create a cylindrical base that was constant across objects. The objects were rotated in 3D in 1° steps about the point on the base closest the edge of the table. Images were rendered in 3DS Max® 2008 and stored in files that could be presented for 100 ms on each trial. Images subtended 29.4° by 22.9° and were viewed at 30 cm. On each trial participants were presented with one of the five objects (random order) at a given orientation and had to report whether they thought the object would fall off the precipice or right itself (YES/NO task). We used a Bayesian adaptive procedure [41] to estimate the psychometric functions relating object orientation to perceived stability for each object, with threshold and slope as parameters of interest and symmetrical lapse rate as a nuisance variable. The CA was the estimated threshold of the function, slope an estimate of the participant's precision. Fewer than 100 trials per object were required to achieve reliable estimates.

### Stimuli and procedure for determining the subjective visual vertical

We measured the subjective visual vertical (SVV) using a variant of the ‘luminous line’ technique [5]–[10]. A simple line probe (2.5°×0.4°) was oriented about a central fixation point (0.38° of visual arc) and briefly presented. For testing the SVV the line was presented in one of 21 orientations (from −50° to +50° in 5° increments), thus the range of lines was always centered about the direction of gravity. The line probe was superimposed on a 36° diameter circular background picture with a neutral grey image. In a control experiment the same background image used in testing the critical angle for leftward falling objects with an upright object was used. All stimuli were displayed for 500 ms and then replaced with a black screen. Participants responded by pressing either a left or right keyboard button using their index and middle fingers of the right hand when upright and left side down, and their left hand when right side down. Participants judged whether the line appeared tilted clockwise or counter-clockwise relative to “the direction in which a ball would fall” (i.e., gravitational vertical). Each stimulus combination was presented ten times using the method of constant stimuli. The order of trial blocks and body orientations was randomized across participants.

A sigmoidal function (Eq. 1) was fit using regression analysis (SigmaPlot v. 9.1) to the proportion of times the line was judged as clockwise relative to gravity as a function of line orientation. The orientation of the line probe where it was equally likely to be judged tilted clockwise or counter-clockwise from gravitational vertical was taken as the perceived vertical.
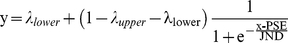
(1)


Where: y =  probability of line being clockwise, λ_upper_ and λ_lower_  = lapse rates for the upper and lower asymptotes of the psychometric function which were each set to be less than 6% [42]–[44], x =  line orientation, PSE  =  point of subjective equality (i.e., SVV); JND  =  just noticeable difference (i.e., standard deviation).
